# The zoonotic pathogen *Wohlfahrtiimonas chitiniclastica* – current findings from a clinical and genomic perspective

**DOI:** 10.1186/s12866-023-03139-7

**Published:** 2024-01-03

**Authors:** Anna Kopf, Boyke Bunk, Thomas Riedel, Percy Schröttner

**Affiliations:** 1 Clinic for Cardiology, Sana Heart Center, Leipziger Str. 50, 03048 Cottbus, Germany; 2 2nd Medical Clinic for Hematology, Oncology, Pneumology and Nephrology, Carl-Thiem Hospital Cottbus gGmbH, Cottbus, Germany; 3 Leibniz Institute DSMZ-German Collection of Microorganisms and Cell Cultures GmbH, Inhoffenstrasse 7 B, 38124 Braunschweig, Germany; 4 German Center for Infection Research (DZIF), Partner Site Hannover-Braunschweig, Braunschweig, Germany; 5 Institute for Medical Microbiology and Virology, Faculty of Medicine and University Hospital Carl Gustav Carus, Technische Universität Dresden, Dresden, Germany; 6 Institute for Clinical Chemistry and Laboratory Medicine, Faculty of Medicine and University Hospital Carl Gustav Carus, Technische Universität Dresden, Dresden, Germany

**Keywords:** *Wohlfahrtiimonas chitiniclastica*, Antibiotic resistance, Arsenic resistance, “One health” approach

## Abstract

The zoonotic pathogen *Wohlfahrtiimonas chitiniclastica* can cause several diseases in humans, including sepsis and bacteremia. Although the pathogenesis is not fully understood, the bacterium is thought to enter traumatic skin lesions via fly larvae, resulting in severe myiasis and/or wound contamination. Infections are typically associated with, but not limited to, infestation of an open wound by fly larvae, poor sanitary conditions, cardiovascular disease, substance abuse, and osteomyelitis. *W. chitiniclastica* is generally sensitive to a broad spectrum of antibiotics with the exception of fosfomycin. However, increasing drug resistance has been observed and its development should be monitored with caution. In this review, we summarize the currently available knowledge and evaluate it from both a clinical and a genomic perspective.

## Background

*Wohlfahrtiimonas chitiniclastica* was first isolated from the larvae of *Wohlfahrtia magnifica* [1], an obligate parasitic fly that causes myiasis by depositing eggs and larvae in wounds of both mammals and humans [2]. *W. magnifica* (Diptera:Sarcophagidae) was first described by Schiner in 1962 [3]. Cells of *W. chitiniclastica* are Gram-negative, strictly aerobic, non-motile rods. The G + C content of the DNA of the type strain DSM 18708^T^ is 44.3 mol% and the major fatty acids are C18:1 and C14:0 [1]. Both catalase and oxidase reaction are positive, while biochemical tests for urease, indole and H_2_S are negative [1]. A key feature is the strong chitinase activity, which may be an indicator of a symbiotic relationship with its host fly and also plays an important role in metamorphosis [1, 4, 5]. To date, several case reports have been published suggesting that *W. chitiniclastica* can cause various diseases in humans as a zoonotic pathogen [6]. Although the pathogenesis of *W. chitiniclastica* has not been fully elucidated, the bacterium is expected to invade traumatic skin lesions through fly larvae, resulting in severe myiasis and/or wound contamination [2, 5–7]. Myiasis is defined as the infestation of living humans and vertebrates with dipteran larvae (maggots) that feed, at least for some time, on dead or living tissues, liquid body substances, or ingested food of the host [2]. In this review, we summarize the currently available knowledge on *W. chitiniclastica* and evaluate it from a clinical and genomic perspective. Since elucidating the significance of *W. chitiniclastica* as a human pathogen is a major focus of our research, we also refer in this review to previously published data from our own scientific work [6, 8]. The aim of this manuscript is therefore to point out gaps in our knowledge on *W. chitiniclastica* by summarizing the currently available data and thus to lay the foundation for further research on this species.

Recently, it could be shown that *W. chitiniclastica* is of importance for both veterinary and human medicine [8]. Although insects currently appear to be responsible for the main transmission, other transmission routes (e.g. through the contact with soil) may be possible as well [6, 9] (Fig. 2). However, these potential routes still need to be clearly elucidated in future studies. The fact that *W. chitiniclastica* harbours a resistance to heavy metals (especially arsenic) could assure this species a survival advantage [9]. In addition, it would also make sense to further clarify if other insects (not only flies) are associated with *W. chitiniclastica* infections. This is of particular interest since *W. chitiniclastica* occurs worldwide and does not seem to be restricted to a particular climate zone [6]. In addition, it can be assumed that *W. chitiniclastica* was not detected in the past due to incorrect identification. According to recent studies, a secure identification of *W. chitiniclastica* is possible using MALDI TOF MS or sequencing of the 16S rRNA gene [6]. With the increasing use of MALDI TOF MS in routine laboratories worldwide, it can be assumed that the number of clinical case reports will increase. This will allow risk factors such as poor hygienic conditions, chronic wounds or diabetes mellitus to be more clearly defined and contribute to a better epidemiological understanding. As there is currently no surveillance system for rare human pathogenic bacteria, clinical case reports play a crucial role in further understanding the epidemiology of *W. chitiniclastica* infections. Since this bacterium is usually part of a polymicrobial infection, future studies need to be conducted to elucidate pathogenicity by exclusively investigating *W. chitiniclastica* isolates. For instance, such studies could address the interaction of *W. chitiniclastica* with the host organism, both in vitro and in vivo (e.g., elucidation of the infection route, interaction with the human skin or the immune system) but also with other microorganisms. As far as sensitivity to antimicrobial agents is concerned, there is a pronounced (possibly primary) resistance to fosfomycin, the genetic basis of which is still unclear. Nevertheless, most isolates seem to be sensitive to quinolones and trimetoprim/sulfamethoxazole. Although *W. chitiniclastica* is currently regarded as a rare pathogen, it is likely that due to the growth of the world’s population (and thus closer contact between humans and animals), the number of zoonotic infections such as the ones caused by *W. chitiniclastica,* will increase [10].

### Members of the genus *Wohlfahrtiimonas*

**Fig. 1:**
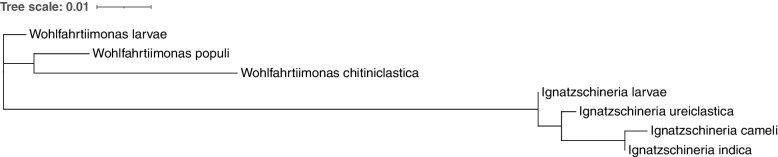
Neighbor-joining phylogenetic tree of partial 16S rRNA gene sequences. Sequences of the type strains were retrieved from GenBank: *W. chitiniclastica* (accession number AM397063), *W. populi* (accession number KT988034), *W. larvae* (accession number JN873914), *I. larvae* (accession number AJ252143), *I. ureiclastica* (accession number EU008089), *I. indica* (accession number EU008088|), and *I. cameli* (accession number LC377575). Phylogenetic tree construction was completed using NGPhylogeny [151] and visualized with iTOL [152]

The genus *Wohlfahrtiimonas* of the class Gammaproteobacteria was first established by Tóth et al. in 2008 [1], and it currently consists of three species [11, 12]. These include *W. chitiniclastica*, *Wohlfahrtiimonas populi* [13] and *Wohlfahrtiimonas larvae* [14] (Fig. 1). Both, *W. chitiniclastica* and *W. larvae* were first isolated from the larvae of dipteran flies [1, 14], whereas *W. populi* was isolated from the bark tissue of the Canadian poplar (*Populus canadensis*) [13]. To our knowledge, neither *W. larvae* nor *W. populi* have been associated with infections in animals or humans. Noteworthy, the genus *Wohlfahrtiimonas* belongs to a distinct lineage close to *Ignatzschineria larvae* which was isolated from first and second larval stages of same fly *W. magnifica* [15, 16]. Like *W. chitiniclastica*, *I. larvae* are considered emerging human and animal pathogens, and have been linked to infections caused by maggot infestation of open wounds [11]*.*

### Zoonotic transmission routes of *W. chitiniclastica*

*W. chitiniclastica* has been described as part of the physiological flora of several fly species such as *W. magnifica* [1], *Lucilia sericata* (Meigen, 1826) (Diptera: Calliphoridae) [17–19], *Lucilia illustris* (Meigen, 1826) (Diptera: Calliphoridae) [20, 21], *Chrysomya megacephala* (Fabricius, 1794) (Diptera: Calliphoridae) [22, 23], *Hermetia illucens* (Linnaeus, 1758) (Diptera: Stratiomyidae) [14, 24, 25] and *Musca domestica* (Linnaeus, 1758) (Diptera:Muscidae) [26, 27]. To the best of our knowledge, flies mainly ensure the spread of *W. chitiniclastica* by depositing larvae in wounds and ulcers of vertebrates, also referred to as myiasis [2, 5]. Of note, the potential application of *I. larvae* and *W. chitiniclastica* in forensic microbiology was recently investigated from necrophagous insect species [20]. The study showed that *W. chitiniclastica* was detectable in all developmental stages of *L. illustris*, with the highest abundances observed in the second and third larval stages [20]. Although further investigations targeting these bacterial species are required to confirm their role as colonization biomarkers in forensic investigations this report highlights the applicative potential of *W. chitiniclastica* in forensic sciences [20].

**Fig. 2:**
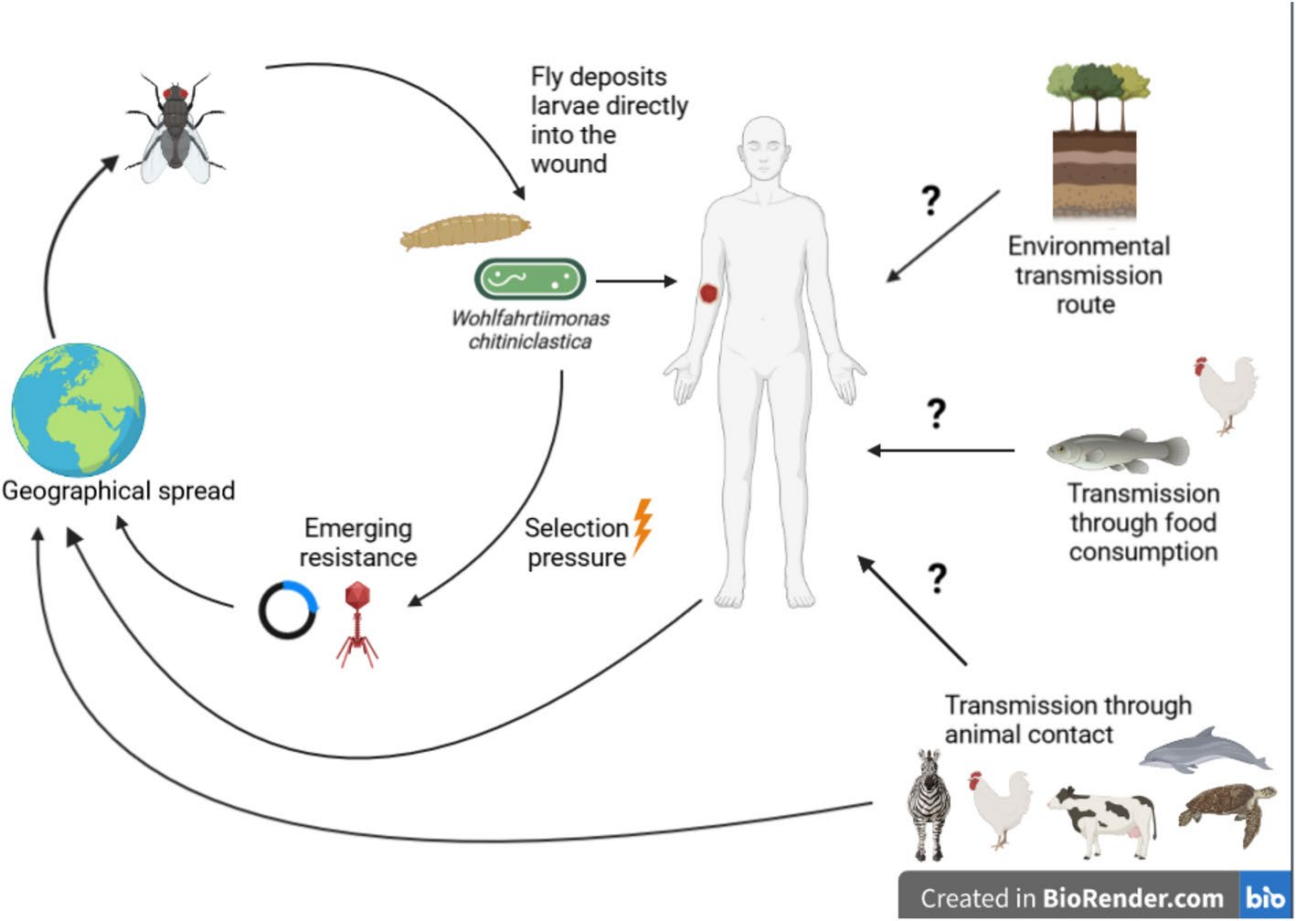
Schematic representation of the possible transmission routes of *W. chitiniclastica*. This figure was created with BioRender.com

In addition to the various fly species, other habitats and zoonotic transmission routes are also conceivable (Fig. 2). *W. chitiniclastica* has also been detected in arsenic-contaminated soil [9], in the pancreas of a zebra [28], in frozen chicken meat [29], poultry chickens in the Noakhali region of Bangladesh [30], in aquatic plants from Egypt [31] and in the human gut microbiome of deceased individuals [32]. In addition to transfer by insects, transmission by contact with the environment or consumption of food also appears to be possible. For example, *W. chitiniclastica* was found abundant in fermented animal and fish-based foods [33] and in chicken meat samples obtained from retail markets [34]. Finally, and more importantly, recent studies indicate that *W. chitiniclastica* may be the cause of several diseases in different organisms. These include marine fish [35, 36], turtles [37], various mammals [7, 38–40] and humans [5, 41], making the bacterium a previously underestimated veterinarian and human pathogen [5, 8]. Initially, *W. chitiniclastica* was described as strictly aerobic by Tóth et al. [1], whereas both *W. populi* and *W. larvae* were described as facultatively anaerobic [13, 14]. Recently, two case studies reported for the first time that the *W. chitiniclastica* strain found in each case also grew under anaerobic conditions [42, 43]. The fact that *W. chitiniclastica* can colonize different species under aerobic and anaerobic conditions should be considered an advantage for the bacterium [8]. The facultative anaerobic lifestyle allows to utilize electron acceptors that are byproducts of host inflammation, thereby increasing its prevalence within the community [44]. On the other hand, it also poses an increased risk for zoonotic transmission, whose dynamic interactions between humans, animals, and pathogens should be considered in the context of the “One Health” approach [8, 45, 46].

### Human infections reported in association with *W. chitiniclastica*

As of June 2023, 43 cases of human infection associated with *W. chitiniclastica* have been published (Table 1). In addition, several case reports relevant to veterinary medicine have also been reported. These include a fatal infection in a deer [7], a hoof infection in a cow [38], a fulminant fish sepsis from India [35], septicemia in a soft-shelled turtle (*Pelodiscus sinensis;* Testudines, Trionychida) [37], interdigital dermatitis in dairy cows [40], evidence of endocarditis infection in a dolphin [39], and preliminary animal infection experiments by Qi et al. suggest that *W. chitiniclastica* is pathogenic to mice [38]. However, since the focus of this review is on the human pathogenic aspect, these reports are mentioned only for completeness. Remarkably, myiasis was not detected in any of these veterinary case reports, supporting the hypothesis that the organism colonizes other ecological niches besides the maggot flora [41].

**Table 1:** Current overview of cases of human infection and colonization with *W. chitiniclastica* as of March 2023. In some cases, antibiotic treatment information was not provided, so there were *marked NP (not provided)*

Case	Year	Age	Gender	Region	Underlying disease(s)/reason for hospital admission	Polymicrobial infection	Insect larvae/infested wounds	Antibiotic treatment	Outcome	Reference
1	2009	60	Female	Marseille, France	Neutroepenia, thrombocytopenia, fatigue	–	yes	Ceftriaxone	Survived	[71]
2	2011	70	Male	Buenos Aires, Argentina	Occlusive peripheral arteriopathy of the lower limbs/sensory impairment	–	–	Ciprofloxacin, ampicillin, ceftazidime, amikacin	Fatal	[61]
3	2015	82	Female	Guildford, UK	Recurrent falls, hypertension, chronic kidney disease, ischemic heart disease, hypercholesteraemia, osteoarthritis/found unconscious	yes	yes	Cefuroxime, metronidazole, clarithromycin, Flucoxacillin	Survived	[72]
4	2015	26	Male	Salt Lake City, USA	Morbid obesity, lymphoedema, cellulitis/progressive gangrenous changes	yes	–	Cefpodoxime	Survived	[56]
5	2015	64	Male	Tartu, Estonia	Gangrene in distal parts of the legs and amputation of the feet/admission due to an accident	yes	–	Amoxicillin/clavulanate	Survived	[31]
6	2015	47	Female	Kubang Kerian, Malaysia	Metastatic colorectal adenocarcinoma, immunosuppressed	–	–	Cefoperazone	Survived	[47]
7	2015	43	Male	Trivandrum, India	Diabetes, deep ulcer, cellulitis, gangrene/progressing gangrenous changes	–	–	Cefoperaxone/sulbactam, cefpodoxime	Survived	[73]
8	2016	17	Male	Cape Town, South Africa	Soft-tissue infection due to an accident	–	–	Ceftriaxone	Survived	[81]
9	2016	72	Male	Honolulu, Hawaii, USA	Stroke, found unconscious	yes	yes	Piperacillin/tazobactam, clindamycin, vancomycin	Fatal	[42]
10	2016	69	Female	Honolulu, Hawaii, USA	Ruptured cerebral aneurysm and right hemiparesis/sacral pain and painful urination	yes	–	Ceftaroline fosamil, meropenem	Survived	[42]
11	2017	41	Female	Columbus, Ohio, USA	Abdominal pain, stage IV right ischial decubitus ulcer, bilateral leg lymphedem, congenital lumbar myelomeningocele causing paraplegiastatus post spinal fixation, extensive sacral decubitus ulcers, obesity, severe lower extremity lymphedema, Arnold Chiari malformation type II with remote ventriculoperitoneal shunt placement and neurogenic bladder with chronic indwelling Foley catheter	yes	–	Vancomycin, cefepime, metronidazol	Fatal	[43]
12	2017	43	Male	Dresden, Germany	Diabetic foot, MRSA	yes	–	NP	NP	[5]
13	2017	78	Male	Dresden, Germany	Diabetes, coronary heart disease, chronic renal failure, venous insufficiency/progressive ulceral disease	yes	–	NP	NP	[5]
14	2017	78	Male	Dresden, Germany	Diabetic foot, ulcus cruris, severe obesity, chronic venous insufficiency, arterial hypertension, chronic heart failure NYHA II, progressive ulceral disease	yes	–	NP	NP	[5]
15	2017	72	Male	Dresden, Germany	Diabetic foot, adiposity, thrombosis, thrombophlebitis, anticoagulation, speech disorder as consequence of a tablet, and alcohol intoxication in suicidal intent	yes	–	NP	NP	[5]
16	2018	57	Male	Washington, USA	Right ankle wet gangrene, chronic cirrhosis, lung atelectasis	–	yes	NP	NP	[54]
17	2018	75	Male	Tokyo, Japan	Squamous cell carcinoma, chronic wounds with maggots	yes	yes	Cefepime, metronidazole	Survived	[48]
18	2018	37	Male	Indianapolis, Indiana, USA	Chronic lymphedema and ulcers of the lower left extremity presented with myiasis of the left foot and leg, myasis	yes	yes	Vancomycin, clindamycin, piperacillin/tazobactam	Survived	[17]
19	2019	54	Male	Melbourne, Australia	Unconscious collapse at home, chronic inflammatory demyelinating polyneuropathy with severe sensory and motor neuropathy, alcohol dependence, and hereditary hemochromatosis	yes	yes	Piperacillin/tazobactam, meropenem, ciprofloxacin	Survived	[74]
20	2019	63	Male	Lexington, Kentucky, USA	Cardiac arrest, anoxic brain injury, foot ucer containing maggots, cirrhosis	yes	yes	Vancomycin, Piperacillin/tazobactam	Fatal	[41]
21	2019	87	Female	Lexington, Kentucky, USA	NP	–	yes	NP	NP	[41]
22	2020	82	Male	Harrisburg, Pennsylvania, USA	Fall at home with asscociated confusion, mitral valve replacement due to mitral stenosis, pe vascular diseases	yes	yes	Vancomycin, cefepime, daptomycin	Survived	[4]
23	2021	70	Male	Fargo, North Dakota, USA	B cell non-Hodgkin lymphoma, chronic left temporal wound	yes	yes	Levofloxacin	Survived	[49]
24	2021	79	Female	Gmunden, Austria	End-stage lung cancer, maltnutrition	–	–	Ampicillin/sulbactam	Survived	[50]
25	2021	63	Male	Baltimore, Maryland, USA	Deep vein thrombosis, chronic venous insufficiency	–	yes	Ceftriaxone, levofloxacin	Survived	[79]
26	2021	63	Male	Brno, Czech Republic	Burn (5%), pediculosis capitis, hepatitis	yes	yes	Amoxicillin, clavulanic acid, metronidazole	Survived	[55]
27	2016	90	Male	Dresden, Germany	Tumorous skin formation (head, neck)	yes	–	NP	NP	[6]
28	2016	82	Female	Dresden, Germany	Renal failure, ulcus cruris	yes	–	NP	NP	[6]
29	2017	79	Male	Dresden, Germany	Diabetic foot, MRSA screening	yes	–	Cefuroxime, levofloxacin	Survived	[6]
30	2017	43	Male	Dresden, Germany	Diabetic foot, ulcus cruris	yes	–	No antibiotic treatment	Survived	[6]
31	2017	78	Female	Dresden, Germany	Diabetic foot	yes	–	No antibiotic treatment	Survived	[6]
32	2017	71	Male	Dresden, Germany	Diabetic foot	yes	–	No antibiotic treatment	Survived	[6]
33	2017	60	Male	Dresden, Germany	Diabetic foot	yes	–	NP	NP	[6]
34	2018	65	Male	Dresden, Germany	Diabetic foot	yes	–	NP	NP	[6]
35	2019	75	Male	Dresden, Germany	Diabetes type 2	yes	–	NP	NP	[6]
36	2019	43	Male	Dresden, Germany	NP	yes	–	NP	NP	[6]
37	2021	50	Female	Stanford, California, USA	Basal cell carcinoma, ulcers, cellulitis	–	yes	Ciprofloxacin, amoxicillin/ clavulanic acid	Survived	[51]
38	2022	48	Male	Baltimore, Maryland, USA	Diabetes type 2, right-foot plantar surface wound, Osteomyelitis	yes	yes	Vancomycin, piperacillin-tazobactam, linezolid, ciprofloxacin	Survived	[80]
39	2022	76	Male	Seoul, Korea	Arterial hypertension, diabetes, diabetic gangrene on distal leg, amputation of three toes	yes	–	Cephamycin, Ampicillin/sulbactam	Survived	[52]
40	2022	53	Male	Mechelen, Belgium	Incomplete spinal injury, chronic wound at the right heel, osteomyelitis	yes	–	Amoxillin/clavulanic acid	Survived	[70]
41	2022	57	Male	Ankara, Turkey	Soft-tissue infection, osteomyelitis, rheumatoid arthritis	yes	–	Cefepime, cefpodoxime	Survived	[75]
42	2022	37	Male	Tulsa, Oklahoma, USA	Diabetes, chronic foot wound, osteomyelitis	yes	–	Piperacillin/tazobactam, ertapenem, daptomycin	Survived	[53]
43	2023	60	Female	Royal Oak, Michigan, USA	Liver cirrhosis, chronic venous insufficiency, malnutrion, chronic wound		yes	Vancomycin, cefepim, cefazolin	Survived	[150]

**Fig. 3:**
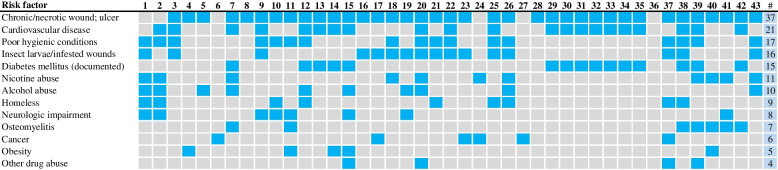
Comorbidities and risk factors for *W. chitiniclastica* infection described in the case report listed in Table 1. Heatmap visualizing the presence/absence of comorbidities in each case. Blue color indicates presence and gray indicates absence. *# refers to the total number*

Human patients with *W. chitiniclastica* infection share some common similarities and risk factors for infection [5, 41]. In particular, patients with chronic/necrotic wounds, cardiovascular disease and poor hygienic conditions were strongly represented, whereas myasis or maggots were only detected in one-third of the patients (Fig. 3). In addition, an association with a history of diabetes, different drug abuse, neurological impairment and osteomyelitis was observed (Fig. 3). It is also worth mentioning that five cases had carcinoma disease as an underlying condition [6, 47–51]. Three of these case studies were part of a polymicrobial infection [6, 48, 49] suggesting that this bacterium may be an opportunistic human pathogen in immunocompromised patients.

**Fig. 4:**
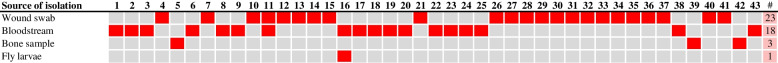
Source of isolation of *W. chitiniclastica* described in the case report listed in Table 1. The red color indicates the evidence shown. *# refers to the total number*

The average age of onset of the patients listed in Table 1 was 62 years. The youngest patient was 17 years old and the oldest patient was 90 years old. Thirty-two patients were male and 11 were female. In general, *W. chitiniclastica* was isolated either from the bloodstream or from wound swabs (Fig. 4). In three cases it was a bone sample [31, 52, 53] and once directly from a fly larva [54]. In most cases, patients received antimicrobial treatment with β-lactam antibiotics, and the vast majority survived the infection (Table 1). Strikingly, *W. chitiniclastica* was often part of a polymicrobial infection in which the bacterium was isolated together with other sepsis-causing pathogens [6, 55]. For example, the bacterium has been described together with *Klebsiella pneumoniae, Acinetobacter lwoffii,* and *Staphylococcus aureus* as a possible source of infection [56]. In other case reports, polymicrobial infection with *S. aureus, Aeromonas* spp.*, Staphylococcus simulans,* and *Bacteroides fragilis* [42] or with *Escherichia coli* [6] was observed. Accordingly, it remains unclear whether *W. chitiniclastica* was the disease-causing pathogen or part of a polymicrobial infection or colonization. Although only rudimentary information is available on the associated microbial community of the *W. chitiniclastica* case studies, similarities with the microbiome described for diabetic foot syndrome can be identified [6]. In particular, the genera *Staphylococcus, Pseudomonas*, as well as *Streptococcus* have recently been described as dominant taxa in chronic diabetic foot ulcers [57–59], while *Proteus* spp. could not be detected in all patients [59]. Interestingly, *W. chitiniclastica* was recently described as a non-dominant part of a microbiome from chronic diabetic ulcers in India [60]. However, there is currently no further evidence of a possible key role in relation to diabetes mellitus and diabetic comorbidities.

Although only a few clinical case reports are available (Table 1), there seem to be no correlation between polymicrobial infection and fatal outcome. In one case, it was reported that monomicrobial sepsis with *W. chitiniclastica* resulted in the patient’s death [61], whereas in another infection, the patient survived [55]. Primarily, the initial health status of the patient on admission to the hospital appears to have an influence on the outcome [5]. Nevertheless, the trend should be further monitored. Especially considering that antibiotic treatment of chronic wounds such as diabetic ulcer does not significantly alter the composition of the microbiome but leads to the selection of resistant pathogens [62, 63]. The presence of multiple resistance genes in different species colonizing an ecological niche in close proximity to each other provides an ideal starting point to promote the formation of multidrug resistance [63]. With respect to *W. chitiniclastica*, this means that the organism can quickly develop drug resistance and may become a serious threat.

### Methods of identification - what works well, less well and why?

Based on the current literature, biochemical approaches such as API (bioMérieux), BD Phoenix Gram Negative Panel (BD Biosystems) or VITEK 2 (bioMérieux) lead to false and misleading results for the identification of *W. chitiniclastica* [5, 6, 31, 41, 61]. Almuzara et al. used the API 20 NE system (bioMérieux, France) which resulted in the identification as *Oligella urethralis* (with 88.5% accuracy) [61]. Similar results were obtained by de Dios et al., where *W. chitinclastica* was identified as *Acinetobacter lwoffii* and *Brevundimonas diminuta* with 98.1 and 88.5% probability, respectively [56]. The BD Phoenix Gram Negative Panel (BD Biosystems, Sparks, MD) lead to a misidentification as *Moraxella* sp. with a low confidence score of 90% [41]. The VITEK 2 system, used by many laboratories worldwide in microbial routine diagnosis [64], also proved to be ineffective in identifying *W. chitiniclastica* isolates [6]. In particular, incorrect identification as *A. lwoffii* [6, 31, 38] or *Comamonas testosteroni* [56] occurred. Noteworthy, most results were above 96%, representing excellent species identification; nevertheless, misidentification was evident in all strains [6, 31, 38, 56]. To the best of our knowledge, the reaction profile of *W. chitiniclastica* is not currently included in the VITEK 2 database [6]. Of note, *A. lwoffii* has been described as part of the physiological skin flora of humans [65, 66] but can also cause severe infections in humans [67]. Therefore, correct identification, including at the best the resistance profile, is crucial to limit the emergence and spread of multidrug-resistant species.

In contrast, MALDI TOF MS, 16S rRNA gene sequencing or *rpoB* analysis have been shown to be safe and reliable identification methods [6, 8]. However, since the *rpoB* approach is not widely established in clinical routine diagnostics yet, MALDI TOF MS and 16S rRNA-based identification most likely remains by far the most frequently used method. It must be assumed that *W. chitiniclastica* was often not detected in the past due to misidentification and that its prevalence in the hospital may have been significantly underestimated [6, 31].

### Geographical distribution and epidemiological aspects

*W. chitiniclastica* has been detected as a zoonotic pathogen in a variety of geographic locations [6]. Initially, the infection was thought to occur only in countries with warm climates [7], but additional human cases have since been reported from a variety of geographic and climatic regions (Table 1). These include 20 cases from Europe, 15 from the United States, 5 from Asia and one each from Africa and Australia (Table 1). Recently, a study surprised with a newly discovered subspecies of *W. chitiniclastica* [6]. It was originally thought to be an adaptation to the human environment and geographic location [6], but recent follow-up studies rather suggests a broad host and environmental range [8, 68]. Considering that *W. chitiniclastica* may not be as rare as originally thought, the host and geographic range might be even wider. This underscores the importance of correctly identifying clinically relevant bacteria to monitor the global spread of infectious diseases and their potential geographic changes [69].

## Antibiotic susceptibility of *W. chitiniclastica*

**Fig. 5:**
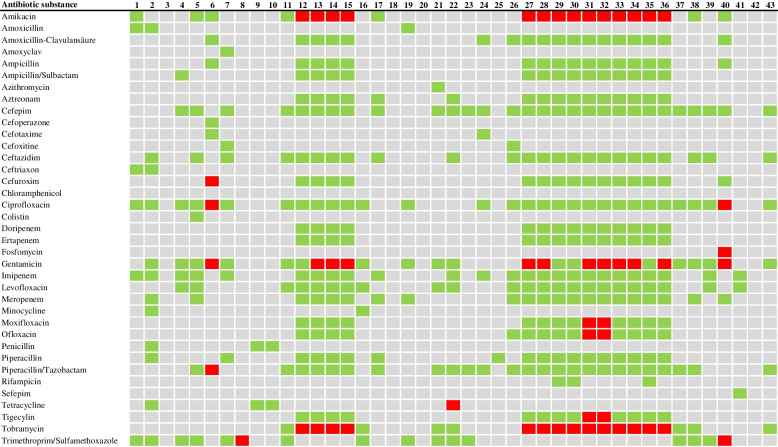
This heatmap presents the antibiotic resistance profiles for the *W. chitiniclastica* strains described in the case reports listed in Table 1. Susceptible isolates are highlighted in green color, and resistant isolates in red. Grey color is shown, if no information is available or no testing has been performed

There have been several case reports on the antibiotic susceptibility testing of *W. chitiniclastica* indicating that the bacterium is generally susceptible to a wide range of antibiotics with the exception of fosfomycin [5, 6, 29, 70]. Figure 5 provides an overview of the resistance profiles of all strains in the 43 cases where human infection was reported. In particular, *W. chitiniclastica* was found to be sensitive to the majority of beta-lactam antibiotics such as penicillin, cephalosporins, and carbapenems. This is consistent with recent case reports in which infections were successfully treated with cephalosporins [4, 49, 71–73] and carbapenems [42, 74, 75]. Furthermore, no specific resistance genes were detected by previous in silico genomic analyses [6, 8]. Of note, one case reported a strain resistant to piperacillin/tazobactam and cefuroxime [47], and a *bla*_*VEB-1*_ gene cassette [68, 76] and *bla*_*OXA-1*_ gene cassette [77] were detected in a rudimentary genomic report of two different *W. chitiniclastica* isolates (Table 2), conferring resistance to ceftazidime, ampicillin, and extended-spectrum β-lactamases (ESBL) resistance to different antibiotic classes [78].

**Table 2:** Overview of all publicly available *W. chitiniclastica* genomes as of March 2023. In addition to information on the host, isolation source, and location, this table provides an overview of the respective genome size and antibiotic resistance genes detected

Strain	Host	Isolation source	Location	Reference	Genome size (bp)	# CDSs	Antibiotic resistance genes
DSM 100374	*Homo sapiens*	Wound swap	Dresden, Germany	[8]	2,079,313	1961	*macA, macB, tehB*
DSM 100375	*Homo sapiens*	Wound swap	Dresden, Germany	[8]	2,103,638	1932	*macA, macB, tehB*
DSM 100676	*Homo sapiens*	Wound swap	Dresden, Germany	[8]	2,139,975	1953	*macA, macB, tehB, tet(H), tet(B)*
DSM 100917	*Homo sapiens*	Wound swap	Dresden, Germany	[8]	2,144,768	1955	*macA, macB, tehB, tet(H), tet(B)*
DSM 105708	*Homo sapiens*	Wound swap	Dresden, Germany	[8]	2,084,087	1969	*macA, macB, tehB, tet(H), tet(B)*
DSM 105712	*Homo sapiens*	Wound swap	Dresden, Germany	[8]	2,133,608	1960	*macA, macB, tehB*
DSM 105838	*Homo sapiens*	Wound swap	Dresden, Germany	[8]	2,069,521	1910	*macA, macB, tehB*
DSM 105839	*Homo sapiens*	Wound swap	Dresden, Germany	[8]	2,123,437	1966	*macA, macB, tehB*
DSM 105984	*Homo sapiens*	Wound swap	Dresden, Germany	[8]	2,120,278	1965	*macA, macB, tehB*
DSM 106597	*Homo sapiens*	Wound swap	Dresden, Germany	[8]	2,131,555	1966	*macA, macB, tehB*
DSM 108045	*Homo sapiens*	Wound swap	Dresden, Germany	[8]	2,090,370	1950	*macA, macB, tehB*
DSM 108048	*Homo sapiens*	Wound swap	Dresden, Germany	[8]	2,074,016	1952	*macA, macB, tehB, abaF*
DSM 110179	*Homo sapiens*	Wound swap	Dresden, Germany	[8]	2,119,644	1965	*macA, macB, tehB*
DSM 110473	*Homo sapiens*	Wound swap	Dresden, Germany	[8]	2,126,147	1970	*macA, macB, tehB*
DSM 18708	*Wohlfahrtia magnitica*	3rd stage larvae of fly	Mezöfalva, Hungary	[1]	1,991,020	1849	*macA, macB, tehB*
SH04	*Chrysomya megacephala*	–	Pudong, China	[22]	2,181,980	2132	*macA, macB, tehB*
BM-Y	Zebra	Pancreas	Shenzhen, China	[28]	2,180,519	2029	*macA, macB, tehB, tet(H), tet(D), ant(2″)-Ia, aac(6′)-Ia, ant(3″)-Ib, bla*_*VEB-1*_
Strain_20	Chicken	Chicken carcass	Rio de Janeiro, Brazil	[29]	2,123,239	1958	*macA, macB, tehB*
ATCC 51249	*Homo sapiens*	Arm	New York, USA	CDC, Atlanta, USA	2,136,105	1973	*macA, macB, tehB*
F6512	*Homo sapiens*	Foot	New York, USA	CDC, Atlanta, USA	2,120,698	1968	*macA, macB, tehB, tet(H)*
F6513	*Homo sapiens*	Leg	New York, USA	CDC, Atlanta, USA	2,115,422	1975	*macA, macB, tehB, tet(H), aph(3″)-Ib, APH* [6]*-Id. sul2, strA*
F6514	*Homo sapiens*	Oral lesion	New York, USA	CDC, Atlanta, USA	2,112,239	1974	*macA, macB, tehB, tet(H), aph(3″)-Ib, APH* [6]*-Id. sul2, strA*
F6515	*Homo sapiens*	Ankle	New York, USA	CDC, Atlanta, USA	2,134,718	2011	*macA, macB, tehB*
F6516	*Homo sapiens*	Arm	New York, USA	CDC, Atlanta, USA	2,071,321	1892	*macA, macB, tehB*
F9188	*Homo sapiens*	Leg wound	Indiana, USA	CDC, Atlanta, USA	2,127,263	1987	*macA, macB, tehB, tet(B), aph(3′)-Ib, aph(3″)-Ib, APH* [6]*-Id, sul2, strA*
G9145	*Homo sapiens*	Wound	Colorado, USA	CDC, Atlanta, USA	2,182,988	2017	*macA, macB, tehB, tet(B), aph(3′)-Ib, aph(3″)-Ib, sul2, strA, cat3*
MUWRP0946	*Homo sapiens*	Wound swap	Kampala, Uganda	[77]	2,080,419	1942	*tet(H), sul2, dfrA1, bla*_*OXA-1*_*, aph(3″)-Ib, aac(6′)-Ib-cr*

The majority of strains showed sensitivity to fluoroquinolones (Fig. 5). This is consistent with recent case studies in which infection caused by *W. chitiniclastica* was successfully treated with levofloxacin [5, 49, 79]. The in silico genomic analysis performed also failed to detect resistance genes specific for fluoroquinolones [6, 8]. Noteworthy, some case reports show resistance to moxifloxacin, ofloxacin [6], and ciprofloxacin [47, 70]. Therefore, when in doubt, levofloxacin should be preferred for planned treatment with fluoroquinolones.

Aminoglycosides such as amikacin, gentamicin, and tobramycin are among the broad-spectrum antibiotics. Several studies have reported that *W. chitiniclastica* is resistant (Fig. 5) [5, 6, 47, 70], and resistance genes have been detected in different genomic studies [8, 77] (Table 2). Consequently, aminoglycosides are not recommended as first-line therapy.

With respect to tetracycline, *W. chitiniclastica* tends to exhibit a diverse antibiotic susceptibility profile (Fig. 5). This observation is also reflected in recent case and research studies. While the majority still appears to be susceptible [6, 31, 41–43, 52, 56, 61, 74, 75, 80], increasing incidence can be observed [4, 6, 47, 70]. Comparative genomic analysis reflected this picture and supported the hypothesis of a rather diverse distribution of tetracycline resistance genes (Table 2) [6, 8, 68, 77]. Noteworthy, these included transposon-encoded *tetR* and *tetC* [8] as well as a plasmid carrying *tetA*(H) [68]. This underscored the hypothesis that the majority of resistance genes in *W. chitinclastica* genomes arose by horizontal gene transfer [8].

Many *W. chitiniclastica* strains are susceptible to trimethoprim/sulfamethoxazole [4, 43, 48, 49, 74]. However, initial resistant strains have been reported from South Africa [81] and Belgium [70], as well as some sulfonamide resistance gene-containing genomes (Table 2) [8, 77]. A plasmid-encoded alteration in dihydrofolate reductase leading to insensitivity to trimethoprim/sulfamethoxazole is particularly common in bacterial pathogens [82] with pronounced geographic differences [83]. For example, trimethoprim/sulfamethoxazole has been shown to be particularly effective against enterotoxin-producing *E. coli* and *Shigella* species in Guadalajara, Mexico [84], whereas resistance levels of > 90% have been observed in Thailand [85]. In the case of the resistant *W. chitiniclastica* isolates [70, 81], it would be conceivable that the organism has expanded its resistance profile through the uptake of a resistance plasmid. In addition, trimethoprim/sulfamethoxazole is a relatively inexpensive drug. Consequently, it has been widely used worldwide for many different infections, which in turn has further promoted the development of resistance [86].

Macrolides, such as azithroymycin, clarithromycin and erythromycin, are antibiotics with bacteriostatic activity. As of June 2023, only Fenwick et al. have reported an azithroymycin-resistant strain [41]. Another research study performed a comprehensive in vitro resistance analysis, and demonstrated relatively high MIC (minimal inhibitory concentrations) values for clarithromycin and erythromycin [6]. However, PK/PD (non-species-related) breakpoints based on the EUCAST (European Committee on Antimicrobial Susceptibility Testing) were not available at that time. Consequently, an evaluation according to the criteria published by EUCAST was not possible, so that no final statement on resistance or susceptibility could be made [6]. In a follow-up study, it was shown that the *macA* and *macB* genes could be detected in all *W. chitiniclastica* genomes that were publicly available at that time (Table 2) [8]. The genes *macA* and *macB* encode for macrolide-specific efflux pumps [87, 88]. Therefore, primary resistance to macrolides is feasible. In addition, *W. chitiniclastica* appears to be resistant to tellurite (Table 2) [8]. This aspect is not surprising since potassium tellurite has been used extensively as an antimicrobial agent in the past and, as a result, many Gram-positive and Gram-negative bacteria have developed resistance [89].

Last but not least, *W. chitiniclastica* is known for its pronounced fosfomycin resistance [5, 6, 29, 70]. Surprisingly, this is not reflected in previous genome studies [6, 8, 22, 28, 29, 77]. Only one genome reveals the presence of the transporter gene *abaF* (Table 2) [8], which confers resistance to fosfomycin [90]. Other well-described fosfomycin resistance genes, such as *fosA, fosC,* or *fomB* [91], do not appear to play a role. It is therefore suggested that *W. chitiniclastica* has an as yet undescribed resistance mechanism to fosfomycin that remains to be discovered [8].

Compared to other pathogens such as *A. lwoffii,* the antibiotic resistance profile of *W. chitiniclastica* is still relatively narrow [92]. Nevertheless, increasing drug resistance has been observed, and its development should be followed with caution (Fig. 5) [8]. For *W. chitiniclastica* infection, levofloxacin and cephalosporins, such as cefepime, appear to be suitable options. However, it should be noted that sensitivity to antibiotics may vary depending on the strain and the specific conditions of infection. Because exposure to many antibiotics leads to tremendous selection pressure, including the spread of resistance [93], it is recommended that clinical isolates be tested for antibiotic susceptibility in order to select the most appropriate antibiotic treatment.

### Striking genomic features of *W. chitiniclastica* and their relevance to adaptation to environmental change

As of June 2023, NCBI lists 28 genomes of *W. chitiniclastica* strains (Table 2), 24 of which have been isolated in the course of human disease [8, 77]. The remaining genomes were isolated from an animal source [22, 28, 29]. A recent comparative genomic study has shed light on various aspects such as virulence factors, mobile genomic elements and pangenomic features [8]. The composition of the pangenome revealed a core genome size of 43%, which is highly conserved compared to other species such as *Clostridium perfringens* (12.6%) [94], *Pseudomonas aeruginosa* (26%) [95], and *K. pneumoniae* (26%) [96], to name a few. Bacteria with a comparatively large core genome often lack a diverse repertoire of virulence and resistance factors and are less able to adapt flexibly and rapidly to changing environmental conditions [95, 97]. This is consistent with recent observations on *W. chitiniclastica*, which are susceptible to most known antibiotics except fosfomycin [5, 6, 29, 70], supporting the notion that members of this species are metabolically conserved compared with others [8]. However, this could change over time. Recent studies have shown that genome-encoded transposons, bacteriophages and plasmids are ubiquitous in *W. chitiniclastica* genomes [8, 29, 68], which could be a key element for the acquisition of new resistance genes [8]. Surprisingly, tetracycline resistance genes in particular were found to be associated with mobile genetic elements such as the *tetA*(H)-carrying plasmid [68] and *tetR* and *tetC* encoded by Tn*10* [8]. Tetracycline is a broad-spectrum antibiotic and is widely used in human and veterinary medicine to treat bacterial infections due to its low price and limited side effects [98–100]. Moreover it has been added to animal feed as a growth promoter [98, 99]. A recent systematic review showed that there is still continuous contamination with tetracyclines in both aquatic and terrestrial animals, leading to selection pressure on antibiotic-resistant bacteria [98] and by that to an alarming rise in antibiotic resistance to tetracycline [101]. As stated above, there is an increasing incidence of drug resistance within the *W. chitiniclastica* clade, most likely acquired through horizontal gene transfer. Noteworthy, all genomes studied to date contain CRISPR-Cas elements and so-called anti-CRISPR proteins (Acr) [8, 29, 77]. The acronym CRISPR stands for “Clustered Regularly Interspaced Short Palindromic Repeats” and is part of the adaptive immune system that enables prokaryotes to recognize and destroy invading foreign DNA [102]. Therefore, in theory, *W. chitiniclastica* should be well equipped against invasive genetic elements including bacteriophages, plasmids, and transposons [103]. On the other hand, Anti-CRISPR (Acr) proteins represent the regulatory counterpart and are thought to be able to inhibit CRISPR-Cas actions [104]. Recent studies have shown that numerous *acr* genes are present in the genomes of various prokaryotes such as *Moraxella bovoculi* or *Pseudomonas* spp. to name a few [105, 106]. Indeed, more than 30% of *P. aeruginosa* strains contain both *acr* and CRISPR-Cas genes [107]. Moreover, a positive correlation between the presence of antimicrobial restiance genes and acr genes has been demonstrated [108]. Currently, the interplay between CRISPR-Cas immunity and ACR activities is thought to be a key element in the adaptation of *W. chitiniclastica* to new environmental conditions [8]. Although the exact function has not been conclusively determined [104], Acr proteins can be expected to slow down the adaptive immune system in *W. chitiniclastica* when needed and enable the uptake of additional resistance genes through genetic mutations and/or horizontal gene transfer between the same or different species to maintain their survivability under the disturbed environmental conditions.

### First insights into potential virulence traits

To date, knowledge about potential virulence traits of *W. chitiniclastica* is limited. Although much research remains to be done, the first interesting findings have recently been published [8]. The ubiquitous genome-encoded presence of several “multidrug efflux systems” and type II secretion systems (TS2) suggests a central role in the pathogenesis of *W. chitiniclastica*. In general, proteins secreted by T2S systems are associated with the destruction of various tissues, cellular damage, and disease. These include proteases, cellulases, pectinases, phospholipases, lipases, and toxins, but secretion of other substances is also feasible [109]. In *Vibrio cholerae*, for example, the T2S system supports secretion not only of cholera toxins and hemagglutinin proteases but also of chitinases [109–111]. *W. chitiniclastica* is known for its distinct chitinase activity [1], which is probably an indicator of a symbiotic relationship with its host fly while also playing an important role in metamorphosis [1, 4, 5]. Thus, involvement of the T2S system in its secretion seems possible [8].

Some *W. chitiniclastica* strains harbor the toxin-encoding gene *relG*, which is known to inhibit mycobacterial growth when expressed independently [112]. Moreover, the ubiquitous presence of the conserved virulence factor B (*cvfB*) suggests a central role in the virulence of *W. chitiniclastica.* Recent studies showed that deletion of CvfB results in reduced virulence in *S. aureus* and decreased production of hemolysin, DNase, and protease [113]. Apart from that, other exotoxin encoding genes appear to be missing or are yet unknown suggesting an alternative virulence profile [8]. Undoubtedly, identifying pathogenesis and toxin encoding genes of *W. chitiniclastica* and its interaction with the host should be further investigated as it could serve as novel targets for drug development. However, there is still a long way to go.

Toxin-antitoxin (TA) modules are ubiquitous in bacteria and are thought to be involved in various physiological processes including virulence [114]. Recently, a *W. chitiniclastica* isolate from China was studied with a novel *bla*_VEB-1_ carrying plasmid [68], which, in addition to antibiotic resistance, also encodes the TA modules RelBE and YefM/YoeB [68]. TA systems that are localized on plasmids are associated with plasmid stabilization and have been shown to increase plasmid maintenance [115, 116]. In contrast, the role of chromosomally encoded TA systems in bacterial physiology has not yet been conclusively elucidated [117]. It is assumed that they have a decisive influence on adaptation to new environmental conditions, improved stress resistance and the stabilization of chromosomal regions [114], which gives the bacteria a considerable fitness advantage [118].

TA modules are also present in several *W. chitiniclastica* genomes [8]. These include the type II TA system YefM-YoeB and PasTI [119]. Previous studies have shown that YefM-YoeB is involved in the colonization of new niches, survival in the host and general stress tolerance [119]. It is therefore conceivable that the TA system is involved in the invasion of new habitats and provides *W. chitiniclastica* with a decisive fitness advantage in the course of a polymicrobial infection. PasTI enables cell formation in the presence of antibiotics and increases the pathogen’s resistance to nutrient limitation as well as oxidative and nitrosative stress [119]. It is worth noting that the function of the *pasT* gene has recently been reannotated based on new experimental evidence [120]. While PasT increases the antibiotic tolerance of pathogens, the function of PasTI as a TA system could not be confirmed [120]. Instead, the putative toxin PasT corresponds to a bacterial homolog of the mitochondrial protein Coq10, which plays a central role in respiratory electron transport as an important cofactor in the ubiquinone-dependent electron transport chain [120]. Therefore, it can currently only be speculated whether the *pasT* gene of *W. chitiniclastica* is primarily involved in virulence and/or energy production. Overall, the role of chromosomally or plasmid-encoded TA systems in bacterial physiology has not yet been conclusively clarified.

### Arsenic resistance genes and their impact on the spread of antibiotic resistance

Arsenic is a natural component of both aquatic and terrestrial habitats. In general, arsenic contamination is relatively low, but the high toxicity of arsenic derivatives is a serious public health concern worldwide [121]. On the other hand, various arsenic compounds have been successfully used as antimicrobial agents in the past and have been used to treat trichomoniasis, malaria, ulcers, and syphilis, as well as a variety of other diseases [122, 123]. This further favored the spread of arsenic resistance genes [121]. Recently, there has also been renewed interest in arsenic as a cancer drug for the treatment of acute promyelocytic leukemia [123, 124]. However, agriculture and industry have primarily contributed to arsenic spread and contamination [125]. In agriculture, animal farming and industrial sectors, arsenic-containing compounds have been used extensively as pesticides and as feed additives, especially in the poultry and swine industries [126, 127]. Roxarsone, for example, was used exclusively in animal farming, particularly in poultry, to promote growth and prevent gastrointestinal infections [125, 128]. Although many arsenic compounds are no longer used, their residues from previous activities are still present, especially in agricultural soils [129] leading to constant selection pressure on bacteria with a tolerance to arsenic [130].

*W. chitiniclastica* was detected in arsenic-contaminated soil in Bangladesh [9]. Recently, a comprehensive genomic study of *W. chitiniclastica* demonstrated the presence of arsenic resistance family genes in all genomes [8]. However, there were some discrepancies with respect to the classical *arsRDABC* operon, and it is possible that *W. chitiniclastica* has a previously unknown or alternative regulatory and/or arsenic tolerance mechanism [8]. In addition to the well-known *arsRDABC* operon, there is an alternative chromosomal arsenic resistance mechanism that has been demonstrated, for example, in *Alcaligenes faecalis* [131]. Here, arsenic is used as a terminal electron acceptor in the absence of oxygen during anaerobic heterotrophic growth [123, 132]. This raises the question of whether the arsenic resistance families encoded in the genome allow growth under anaerobic conditions in the presence of arsenic. Interestingly, *W. chitiniclastica* was described as strictly aerobic when it was first described [1], while both *W. populi* and *W. larvae* were described as facultatively anaerobic [13, 14]. Recently two case studies reported that the respective *W. chitiniclastica* strain also grew under anaerobic conditions [42, 43]. This allows initial speculation about further metabolic properties of *W. chitiniclastica* that have not yet been described, according to which strains of this species can be characterized mainly as facultative anaerobes. However, further experimental studies in combination with in silico genome analyses, at best including targeted genetic manipulations, are required to confirm this hypothesis.

At first glance, bacterial arsenic resistance appears to be of little interest to human medicine despite that fact that arsenic resistance genes are widely distributed in human pathogens [123]. Although improper use of antibiotics is known to favor the selection and spread of antibiotic resistance [133], metal contamination can also promote the spread of antibiotic resistance through multifactorial coselection mechanisms [133–135]. It has recently been shown, that the use of heavy metals for growth promotion in poultry farms resulted in the coselection of mobile genetic elements and antimicrobial resistance genes [135]. Often, the corresponding genes are encoded in a common resistance gene cassette on the same mobile genetic element such as transposons or plasmids [123]. For example, the sulfonamide resistance gene *sul2* has been detected together with the arsenic resistance genes *arsA, arsB, arsC, arsD,* and *arsR* [136]. In fact, arsenic-polluted environments have been described as contributing to the co-selection of antimicrobial resistance genes and mobile genetic elements [125]. These include β-lactamases *(bla*_CMY_/*ampC)*, macrolides (*erm35*), MLSB (*erm(F)),* tetracyclines *(tet(B))*, aminoglycosides (*aadA/aacC)*, and transposons (Tn*21*/Tn*22*/Tn*24*/Tn*614*) [125, 137–139]. This has been demonstrated in numerous human pathogens [123], such as *Campylobacter jejuni* [140], *S. aureus* [141], and *K. pneumoniae* [142], to name a few. In all cases, there is a selection advantage for bacterial survival. Unlike antibiotics, metals do not degrade in the environment and their presence could therefore represent a long-term selection pressure [134]. Although the overuse of antibiotics is one of the main driving force of antibiotic resistance, arsenic-polluted environments have been described to contribute to the co-selection of genes for antimicrobial resistance [125]. For example, the presence of arsenic and other metals in a Chinese poultry production was recently shown to have a stronger impact on the composition of metal tolerance and antibiotic resistance genes than some antibiotics [135]. Interestingly, a positive correlation was found between arsenic concentrations and the resistance genes for aminoglycosides [*aac* [60]- *Ia*], macrolides (*erm35*), bacitracin (*bacA*) and tetracycline (*tet* genes) [135]. Another study from rural Bangladesh showed that co-resistance to arsenic and antibiotics in *E. coli* was more pronounced in areas with high arsenic levels than in areas with low arsenic levels [143].

In the context of the development of antibiotic resistance and its far-reaching consequences, arsenic resistance in *W. chitiniclastica* is of critical importance and should be considered in the development of strategies to combat antibiotic resistance. Again, it would be useful to seek interdisciplinary collaboration based on the “One Health” concept to rapidly identify environmental conditions with increased risk of metal-induced coselection and to counteract the spread of antibiotic resistance genes [123, 133, 144, 145]. The focus should not only be on the restrictive use of antibiotics. The positive association found between arsenic exposure and antimicrobial resistance in arsenic-contaminated areas is a major public health concern and warrants increased efforts to reduce arsenic exposure [143]. There is an urgent need to develop guidelines on national as well as international level to control the rampant and uncontrolled use of numerous chemical substances including arsenic-containing compounds. In addition, it is particularly necessary to launch a far-reaching awareness-raising campaign for the general public by providing targeted information about the risks of improper and unjustified use of antibiotics and metal-containing compounds, and show what each individual can do to prevent the development of resistant bacteria.

### The relevance of genomic studies for understanding infectious diseases

In recent years, more and more studies have been published showing the benefits of investigating bacterial genomes for diagnostic microbiology and how genomic comparisons make it possible to significantly reduce analysis times and increase the accuracy of the results [146]. The most important applications are the investigation of antimicrobial susceptibility, the disclosure of virulence factors, surveillance and the clarification of outbreaks in hospitals, but also the assignment of a clear species affiliation of an isolate [146]. The phenotypic expression of resistance in Enterobacterales for example may indicate the presence of carbapenemase, although it is based on efflux pumps or changes in membrane permeability and therefore has no direct impact on hospital hygiene measures [147, 148]. The relevance of the correlation between phenotypic expression of antimicrobial susceptibility and genomic data can also be illustrated by our own studies on *W. chitiniclastica*. The postulation of a jet unknown resistance mechanism for fosfomycin was only possible by comparing the (high) MIC values with the genetic databases for resistance genes [6]. In addition, genomic investigations can reveal previously unknown biovars with a potential clinical impact. Antonation et al. for instance were able to show that a certain clade of african *Bacillus cereus* strains exhibited virulence properties of *Bacillus anthracis* by harbouring the corresponding virulence plasmids. The authors thus named the new biovar *Bacillus cereus* biovar anthracis [149]. This is significant because *B. cereus*, in contrast to *B. anthracis*, usually causes only transient and mild intoxications or infections. Although this biovar has not yet appeared in a human medical context, this cannot be ruled out in the future due to worldwide travel, but also due to the fact that *B. cereus* is capable of spore formation. Regardless, the investigations of bacterial genomes will allow us to gain a deeper understanding of the distribution and diversity of rare pathogens and their impact on public health and wildlife populations [149].

## Conclusion

This review provides an overview of the current knowledge and perspectives of *W. chitiniclastica* from a clinical and genomic perspective. This bacterium has recently been described as a rare but potentially emerging human pathogen whose occurrence is associated with, but not limited to, certain flies. However, because conventional biochemical identification tools can be unreliable and misleading in identifying this organism, this species may be even more widespread than previously thought. Cases of *W. chitiniclastica* infection usually have a number of characteristic underlying conditions. In particular, these include poor hygienic conditions and chronic wounds. In addition, *W. chitiniclastica* is often found to be part of a polymicrobial infection and is considered an opportunistic pathogen in immunocompromised patients. The presence of multiple resistance genes in different species colonizing an ecological niche in close proximity to each other provides an ideal starting point to promote multidrug resistance formation. Although *W. chitiniclastica* is generally sensitive to most classes of antimicrobial agents, increasing drug resistance has been observed. This trend should be critically monitored and evaluated in the context of the “One Health” concept. Deciphering virulence systems and pathogenicity will be the next critical step in understanding *W. chitiniclastica* in order to develop strategies to control its spread.

## Data Availability

No original data are analysed in this manuscript. The genome data mentioned in this manuscript are available via NCBI.
